# Automated Segmentation of Optical Coherence Tomography Images of the Human Tympanic Membrane Using Deep Learning

**DOI:** 10.3390/a16090445

**Published:** 2023-09-17

**Authors:** Thomas P. Oghalai, Ryan Long, Wihan Kim, Brian E. Applegate, John S. Oghalai

**Affiliations:** 1 Department of Electrical and Computer Engineering, University of Wisconsin-Madison, Madison, WI 53706, USA; 2 Caruso Department of Otolaryngology-Head and Neck Surgery, University of Southern California, Los Angeles, CA 90033, USA

**Keywords:** deep learning algorithm, tympanic membrane, Tensorflow, optical coherence tomography, convolutional neural network

## Abstract

Optical Coherence Tomography (OCT) is a light-based imaging modality that is used widely in the diagnosis and management of eye disease, and it is starting to become used to evaluate for ear disease. However, manual image analysis to interpret the anatomical and pathological findings in the images it provides is complicated and time-consuming. To streamline data analysis and image processing, we applied a machine learning algorithm to identify and segment the key anatomical structure of interest for medical diagnostics, the tympanic membrane. Using 3D volumes of the human tympanic membrane, we used thresholding and contour finding to locate a series of objects. We then applied TensorFlow deep learning algorithms to identify the tympanic membrane within the objects using a convolutional neural network. Finally, we reconstructed the 3D volume to selectively display the tympanic membrane. The algorithm was able to correctly identify the tympanic membrane properly with an accuracy of ~98% while removing most of the artifacts within the images, caused by reflections and signal saturations. Thus, the algorithm significantly improved visualization of the tympanic membrane, which was our primary objective. Machine learning approaches, such as this one, will be critical to allowing OCT medical imaging to become a convenient and viable diagnostic tool within the field of otolaryngology.

## 1. Introduction

The tympanic membrane (TM), also known as the eardrum, is vital to hearing because it converts sound pressure waves in air into mechanical vibrations. These vibrations are then passed through the ossicular chain (the three middle ear bones) into the cochlea, where the vibrations are transduced into neural signals which our brain interprets as sound [1,2]. The structure of the TM is important for normal hearing. Many diseases cause hearing loss by altering the TM, such as perforations [3–7], retraction pockets [8–11], and cholesteatoma [11–13]. Other ear diseases cause hearing loss by affecting the middle ear space, which lies directly behind the TM, such as ear infections, middle ear fluid, or tumors of the middle ear. Thus, proper evaluation of the ear is critical for the diagnosis and treatment of these conditions [14]. Current medical management involves using an otoscope [15], which uses a small light with a magnifying glass to visualize the TM deep within the ear canal. However, this method of examining the ear is limited in the information it provides. It does allow the physician to see the surface of the TM. It can sometimes permit a limited ability to view the middle ear space and its underlying structures through the translucent nature of the TM. Sometimes, the illumination can be inadequate, making otoscopy challenging. Furthermore, accurate otoscopic evaluations require extensive training and familiarity with the appearance of normal anatomy and pathology on examination, with conclusions often depending based on physician experience. Thus, making the clinical diagnosis of an ear infection is surprisingly fraught with difficulty, and it is common for this to be missed by routine clinical exam [16,17].

A potential adjunct to otoscopes that could provide additional anatomical and functional information on the TM and middle ear space to improve the diagnosis and management of ear disease is the use of Optical Coherence Tomography (OCT) [18]. OCT is an interferometry-based non-invasive imaging modality in which laser light is focused into a biological tissue of interest. The back-reflected light is analyzed to provide a depth profile of the tissue (Z-direction), which is comparable to how ultrasound works. By scanning the laser beam in the X and Y directions, 3D morphological images of the sample are obtained. OCT is already in wide clinical use in ophthalmology to help in the diagnosis and treatment of various eye pathologies [19,20]. It is now being explored for applications within the field of otolaryngology, commonly known as Ear, Nose, and Throat [21]. Our research group has been using it to study the ear [22–24]. The theoretical benefit of OCT for imaging the human ear is that it provides a 3D image of the TM, as opposed to only the 2D surface view that one gets from looking through an otoscope. Thus, it should improve the diagnostic sensitivity for ear disease and ultimately permit machine learning approaches to automate the diagnostic process.

**Figure 1.**
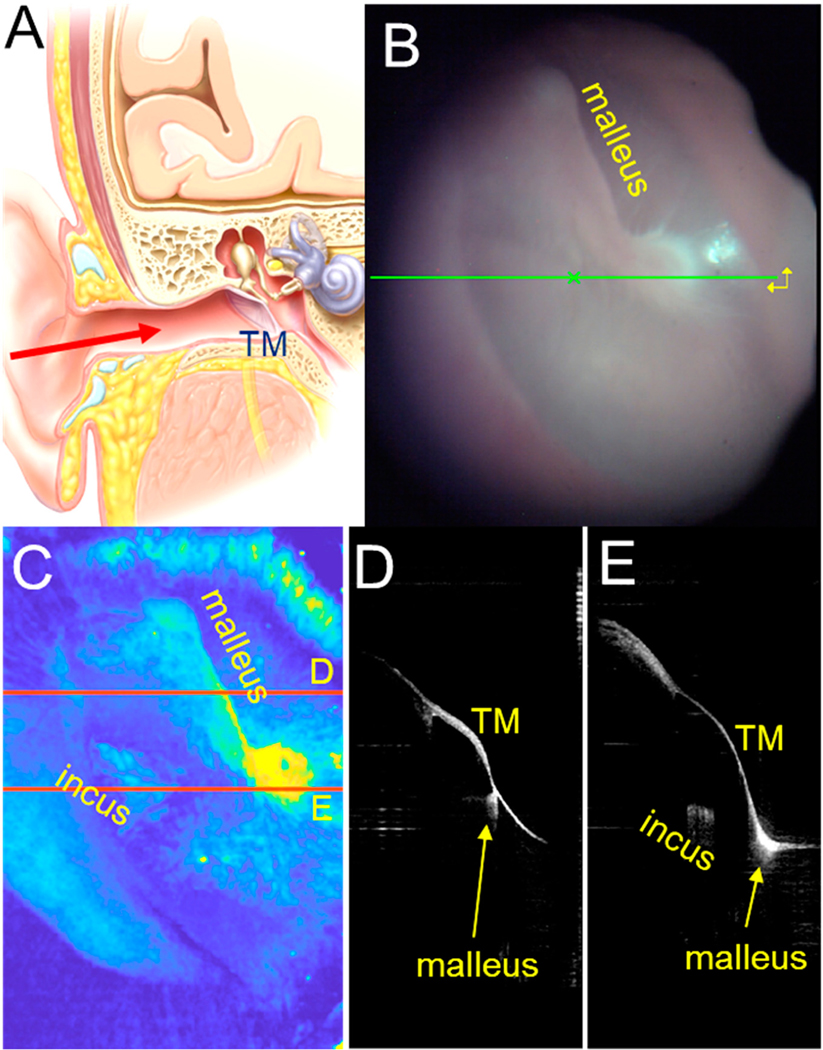
OCT images of the human ear. (**A**) Illustration of the ear. The tympanic membrane (*TM*) is visible by looking down the ear canal (red arrow). (**B**) A video image of the TM, looking through an otoscope. The malleus (the first of the three middle ear bones) is visible along its attachment to the TM. The green line helps the users know where the line scan is being performed. (**C**) A summed voxel projection created from an OCT image stack of a human TM. It has been pseudocolored so that yellow indicates higher reflectivity and dark blue indicates lower reflectivity. The malleus is visible. Also, the incus (the second middle ear bone) is visible because it can be detected by OCT. The red lines illustrate the X-Z slices shown in *D* and *E*. (**D,E**) X-Z slices from the image stack collected in the locations indicated by the two red lines in *C*. Note the thin curved appearance of the TM. The malleus and incus can also be seen under the TM.

We developed a handheld OCT device that provides 3D volumes of the ear [25] that allows one to view beyond the surface level of the TM and image deeper structures often affected by middle ear pathologies (Figure 1) [26]. However, the large, high-resolution image volume stacks are difficult to interpret. It is challenging to distinguish between the various anatomic structures within the ear (e.g., the TM, the ossicles, the cochlea), artifacts that stem from strong tissue reflections that saturate the detector, and fixed pattern artifacts that come from reflections within the optical device itself. Furthermore, the orientation of the image will vary depending on the way the physician holds the device relative to the patient and the patient’s individual anatomy. Therefore, while it seems like it should be simple to collect a volume scan of a patient’s ear and look at the results immediately, all these factors make it difficult to expeditiously interpret the imaging and make a clinical decision at the point of care. Here, we showed how an artificial intelligence approach based on a deep learning algorithm can be used to address this limitation. We developed an algorithm to quickly locate and segment normal TM anatomy. The main contribution of this algorithm is that it removes artifacts from the OCT images, leaving behind the key structure of interest, which is the TM. Deep learning algorithms for segmentation and diagnostics are starting to be used within the medical field [27–31]. This algorithm, thus, represents an early stage in AI-automated diagnosis of ear disease.

## 2. Methods

### 2.1. Patient Dataset

This study was approved by the institutional review board at the University of Southern California (protocol HS-17–01014). We collected data from sixteen participants with no known history of ear pathology from the senior author’s clinic. All participants had normal ear exams on the day of OCT imaging. Participants ages ranged from 26 to 45 years of age.

OCT volume scans of the posterior half of the tympanic membrane were collected using the same techniques as described in our previous studies [25,32]. Briefly, the participant was seated in an exam chair, and the speculum of the hand-held OCT device was inserted into the ear canal. The TM was brought into focus and the volume scan was collected. This took about 20 s per ear. We used a mixture of both right and left ears for this study.

Each OCT volume scan consisted of 417 X-Z slices (B-scans) that were 417 pixels wide by 669 pixels deep, with a total imaging range of 8 mm (X) by 8 mm (Y) by 12.83 mm (Z). The optical resolution of our OCT device was 35 μm in the X, Y, and Z dimensions, and so, the data were oversampled. Scans were anonymized and then used for this research. We used eight of the volume scans for training the models and the other eight volume scans for validating the finished algorithm (i.e., 8 volume scans * 417 B-scans per volume scan = 3336 images for training and another 3336 images for validating).

### 2.2. Overview of Our AI methodology

**Figure 2.**
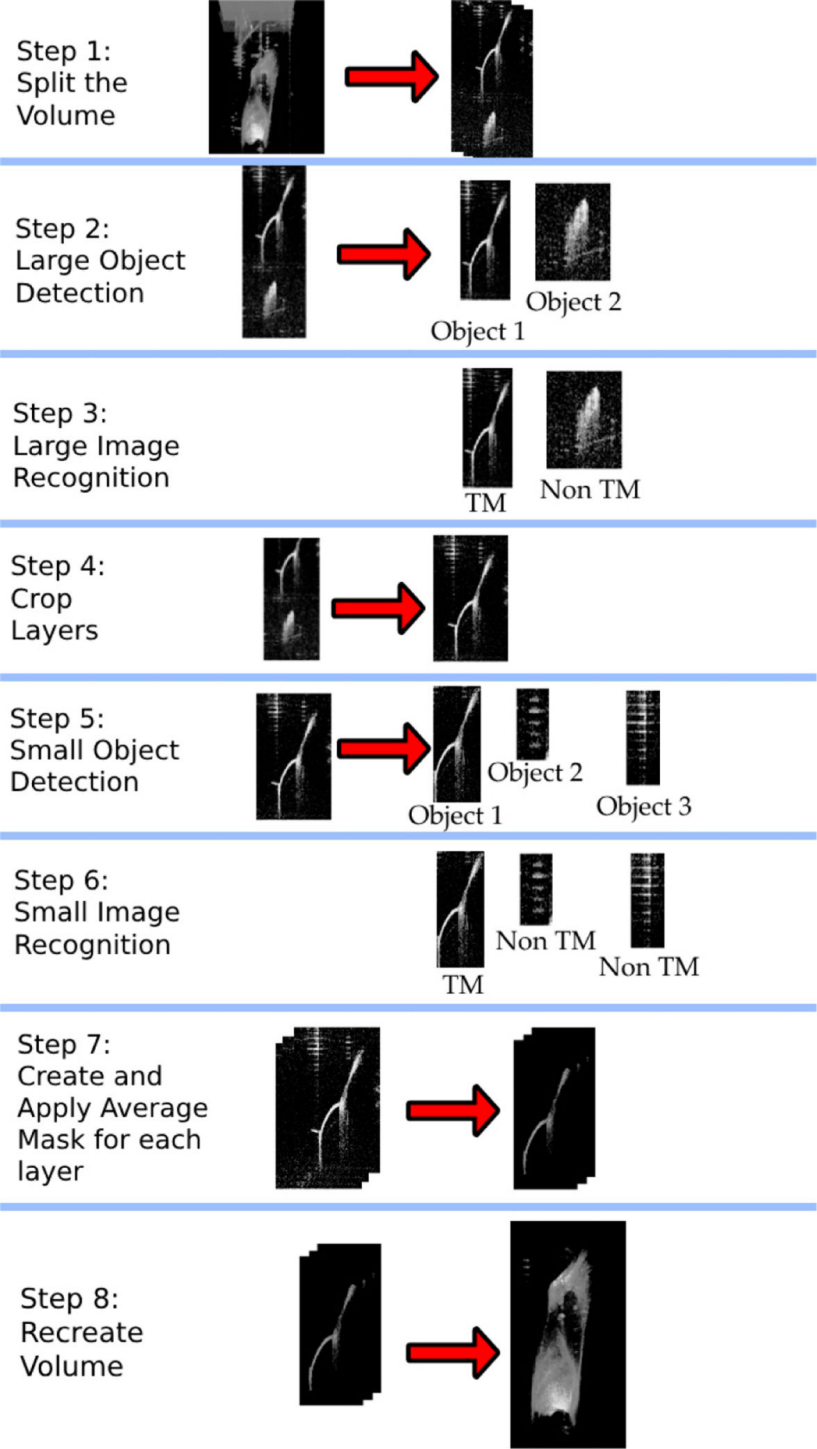
The sequence of steps in our deep-learning-based AI algorithm.

Creating and training our AI methodology involved multiple sequential steps (Figure 2). First, large objects within each X-Z slice of the volume stack were detected using the *large object detection algorithm*. We then manually looked at each object and manually classified it as either TM or non-TM. Next, these large objects were used to train the *large image recognition algorithm* using machine learning. Then, all large objects classified as TM were then re-evaluated using the *small object detection algorithm*, which detected smaller objects that were near the TM. Each new object was then manually classified as either TM or non-TM. Finally, these new smaller objects were used to train the *small image recognition algorithm* using machine learning.

Once trained, using our two-stage AI methodology (first detecting large objects then detecting small objects) was completely automatic and did not require any user involvement. A 3D image stack was sent to the function and a 3D image stack containing only the segmented TM was returned. The entire algorithm was implemented on a PC-compatible desktop computer with an AMD Ryzen 5 3600X 6-Core Processor CPU running Python code. All code and datasets are available for download on our GitHub site [33]. Each step in our AI methodology is explained in detail below.

### 2.3. Large Object Detection Algorithm

The goal of the large object detection algorithm is to distinguish large structures within the collected images. Each 3D volume image collected by our OCT device was a tiff stack of 417 image slices (B-scans). Each slice represented a 2D cross-section of the volume with the X dimension on the horizontal axis and the Z dimension on the vertical axis. The Y coordinate of each image represents its order in the stack. Each image slice was analyzed sequentially.

First, the image was thresholded to convert each pixel to either black or white, to highlight structures. A higher threshold value resulted in fewer potential objects. This made the process faster, but also lowered accuracy and increased the chances of cropping out portions of the TM that were less intense. Then, the image was blurred to reduce the chance of one object being split up into multiple smaller objects. However, increasing the blur value too much increased the risk of artifacts or middle ear structures appearing to be part of the TM. Next, each large aggregation of white pixels was identified and contour detection was performed. Finally, the size of each aggregation was calculated. If it was higher than the minimum size we defined, the aggregation of white pixels was termed an “object” and the coordinates were saved. A higher minimum size requirement sped up the algorithm but could miss some of the TM objects. Thus, there were three parameters we considered when detecting objects: the threshold value, the blur value, and the minimum size. We iteratively varied these parameters until we achieved a decent balance between accuracy and speed.

From the images collected from the eight participants used for training, this algorithm identified roughly 45,000 large objects. We then went through these manually one-by-one and found that ~43,000 objects were non-TM and ~2000 objects were TM. The reason that there were fewer TM objects was that about half of the slices within each volume stack contained the TM and this would be identified as only one object. In contrast, each slice had multiple distinct artifacts, each identified as separate objects.

### 2.4. Small Object Detection Algorithm

Within the 2000 TM objects, we found that there were many artifacts that blurred together with the TM. To overcome this, we implemented a second small object detection algorithm, with the goal being to sort through only the TM objects and remove artifacts within them. The benefit of having this two-stage approach was that the more time-intensive process of separating TM from nearby non-TM objects could be focused on fewer and smaller images (i.e., just the 2000 TM objects).

First, the TM objects were selected from the original image data. These were rectangular-sized images with a corner position, length, and width that came from the TM objects detected with the large object detection algorithm. The same sequence of steps was then used, threshold, blurring, and aggregation size, to detect all objects within the image. However, the parameters were different than what was used for the large object detection algorithm, so as to detect most white pixels within the image. Thus, many more objects were found within this single TM object. We found that a good tradeoff in accuracy versus speed was to set the threshold value lower, the blur value lower, and the minimum size just slightly higher. This is due to the fact that more small objects will be detected. By increasing the maximum size threshold, the program will remove more objects before identifying them and complete its process more quickly.

Again, we went through each newly detected object and manually classified each one as either TM or non-TM. Thus, the 2000 TM objects could be broken down and re-evaluated into ~1000 TM objects and ~9000 non-TM objects.

### 2.5. Image Recognition Algorithms

**Figure 3.**
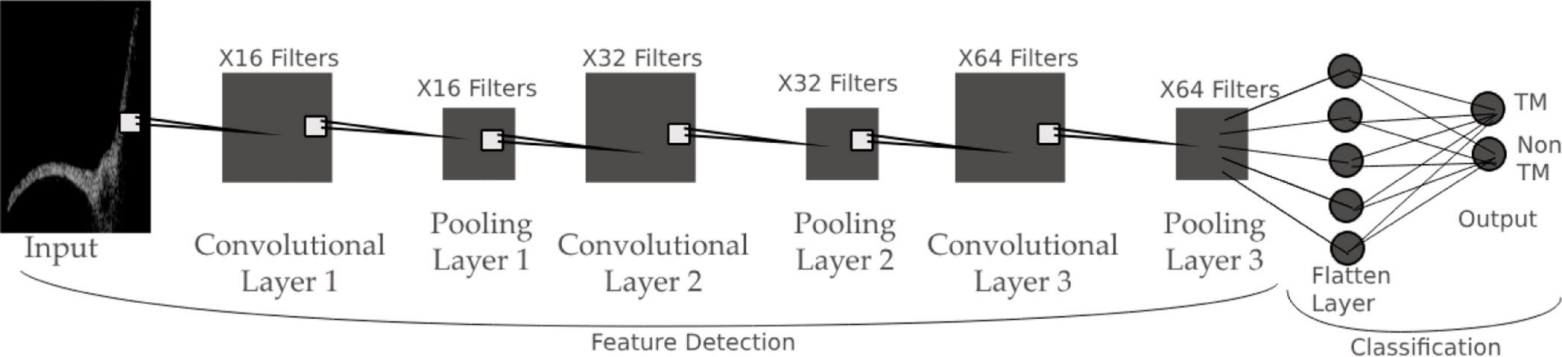
The structure of the convolutional neural network.

We used TensorFlow to create two neural network-based image recognition algorithms [13]. Both algorithms used the same convolutional neural network approach (Figure 3). The only difference was that the large image recognition algorithm was trained on objects from the large object detection algorithm. Similarly, the small image recognition algorithm was trained on objects from the small object detection algorithm.

Data augmentation was performed by rotating each TM object in varying amounts, thus generating additional TM objects for training both neural networks. Data augmentation allowed the model to better identify the TM more accurately; however, it does carry the risk of overfitting the model to the training data [34–36].

### 2.6. 3D Reconstruction Algorithm

Once all the slices have TM and non-TM objects classified using the two-stage approach, the 3D TM object needs to be reconstructed. To carry this out, the TM object locations were used as a mask in order to remove all other objects from each slice. The algorithm then reconstructed the volume so that it could be viewed as a 3D object.

## 3. Results

### 3.1. Model Results

After training the two-stage algorithm on the data from the eight human participant, we tested it on eight additional volume sets collected from different human participant. This demonstrated that the TM was correctly identified in 95% of the objects. This was determined by going through each individual object identified from within each slice afterward by hand and assessing the accuracy of the detection algorithm. The algorithm only required the 3D volume, with no human intervention necessary. Using our PC-compatible desktop computer, analyzing all eight image stacks took about 66 min total, meaning that the average time to segment the TM from one image stack was 8.25 min.

**Figure 4.**
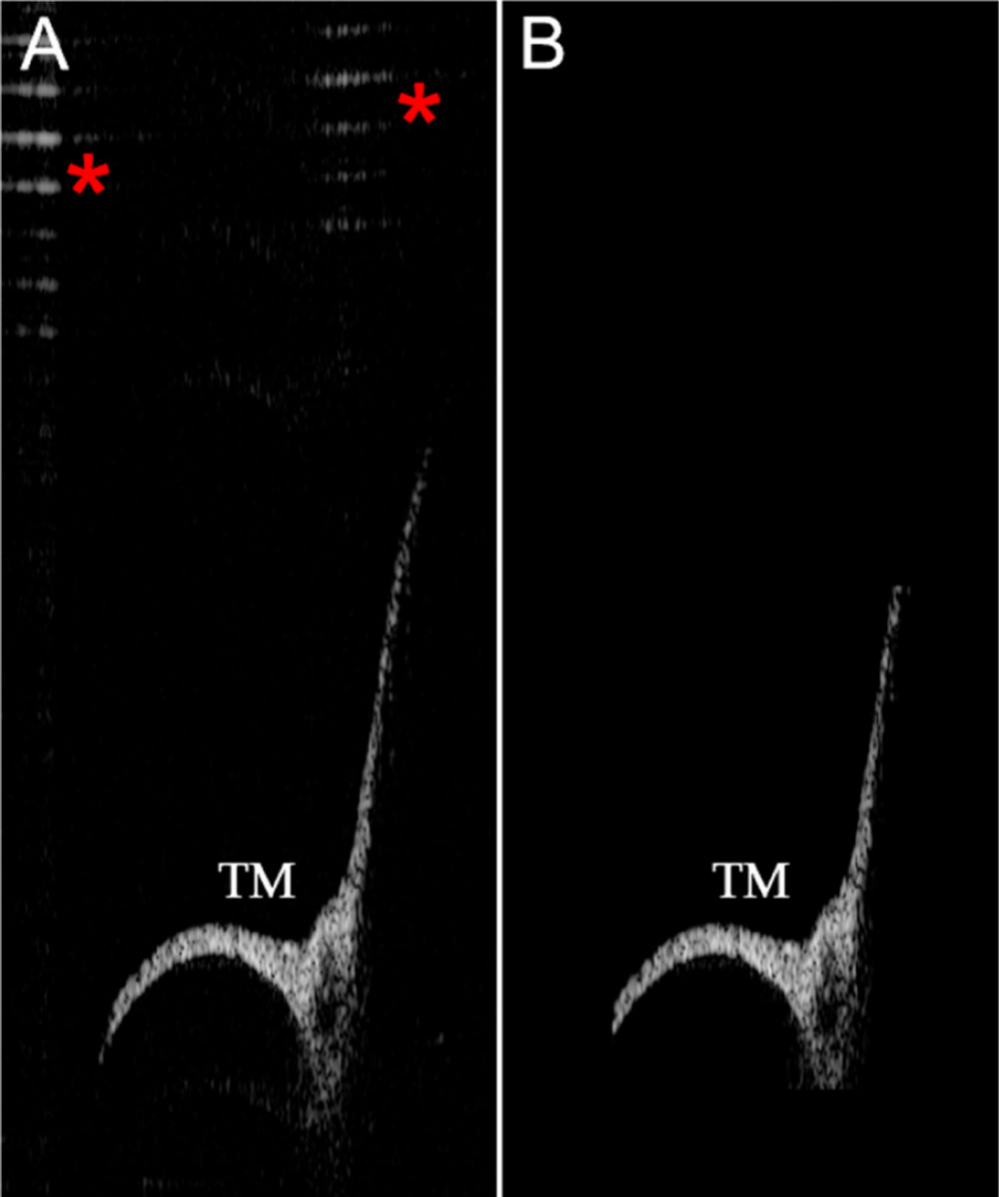
Representative example demonstrating how the algorithm segments out the TM and removes artifacts. (**A**) One slice from the original image. Artifacts are indicated (*red asterisks*). (**B**) After automatically segmenting the TM, the artifacts have been removed.

**Figure 5.**
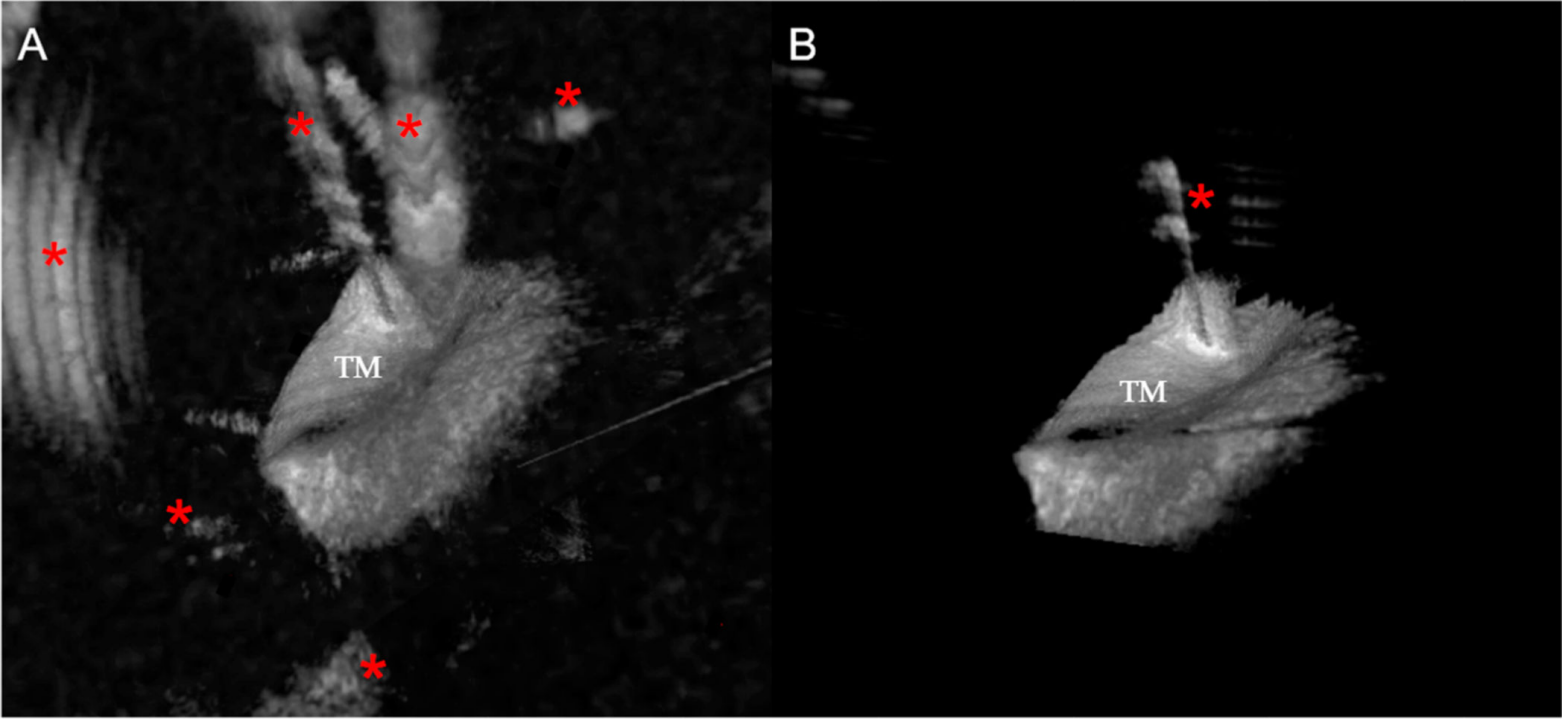
Representative example of 3D reconstructions of one OCT volume of the tympanic membrane. (**A**) Before and (**B**) after running the algorithm to segment out the tympanic membrane (*TM*). Artifacts (*red asterisks*) were either completely removed or greatly reduced in size after segmentation.

The representative examples of single slices after segmentation revealed isolated TMs, with most artifacts removed (Figure 4). After every slice was parsed for the TM, the algorithm reconstructed the volume so that it could be viewed as a 3D object (Figure 5).

**Figure 6.**
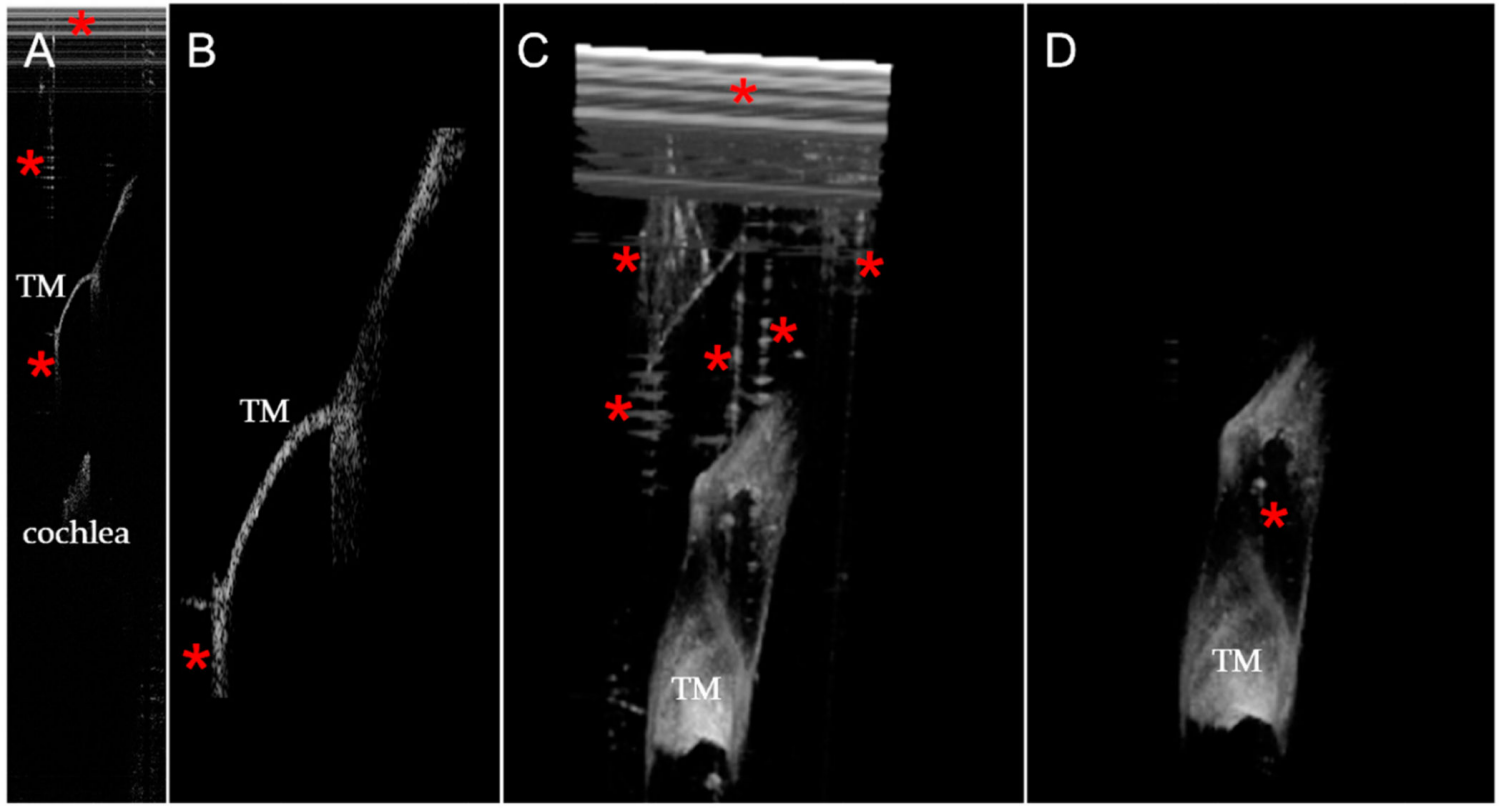
Examples of artifacts that could and could not be removed from the TM. (**A**) One slice from an original volume stack. Artifacts are identified (*red asterisks*). (**B**) The TM object from the same slice. Note that while the cochlea and most of the artifact was segmented out, some residual artifact remained at the bottom of the TM. (**C**) The original 3D volume projection contained many artifacts. (**D**) The segmented 3D volume projection of the TM was much cleaner. However, there was still some artifact within the TM that could not be segmented out.

The two most common instances of artifacts across hundreds of images were a set of white lines at the top of the image (due to background fixed pattern noise) and vertical lines (due to strong tissue reflections). Our algorithm provided a reasonably good approach to removing both artifacts (Figures 5 and 6).

### 3.2. Model Details and Rationale for Overfitting

**Figure 7.**
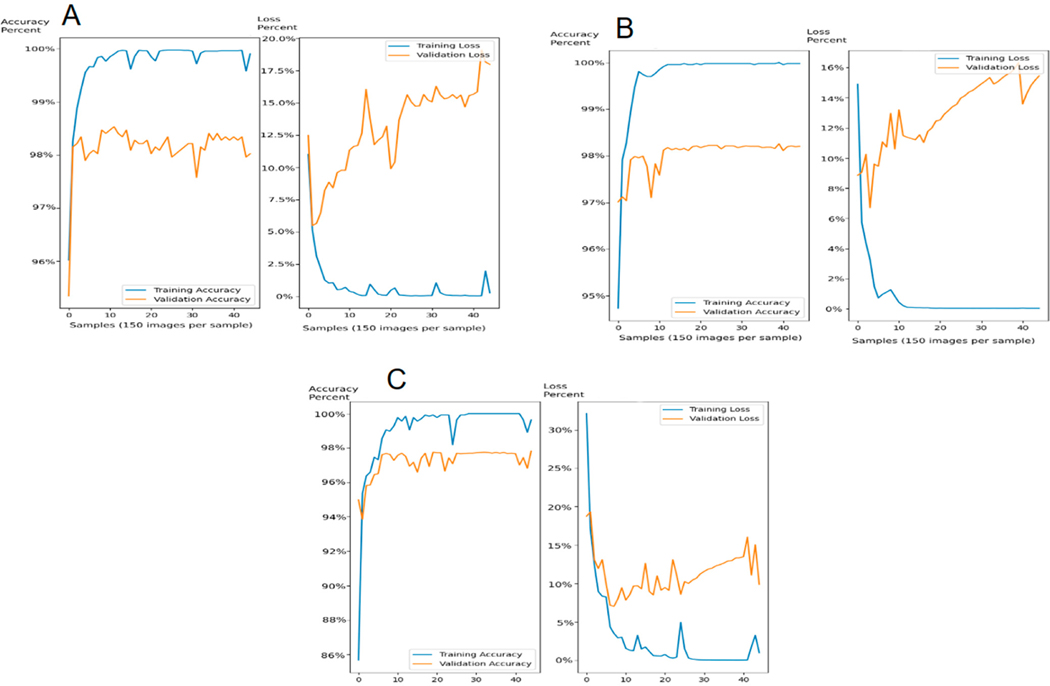
Accuracy and loss during training and validation. (**A**) The large image recognition model. (**B**) The small image recognition model. (**C**) The small image recognition model trained with only 25% of the data.

As described earlier, the accuracy of the model on new data (i.e., the image stacks from the eight subjects used for validation) was 95%. To achieve these results, we evaluated the training accuracy and loss for both the large and small image recognition algorithms (Figure 7). Training accuracy and loss were computed by TensorFlow automatically during the training process, and so, only image stacks from the eight training participant were used to generate these plots. The training accuracy corresponded to the percent identified correctly within the subset of the volume stacks used to train the algorithm. The validation accuracy represents the percentage of images that the program correctly identified within the remaining volume stacks. Thus, training accuracy will always be higher than validation accuracy. The training loss represents the penalty involved in failing to identify an image correctly and goes up with overfitting the models.

The *x*-axis on each graph was measured in samples. Each sample represents one set of training data, and we used 45 samples per trained model. Each sample contained 182 images, and so, the program plotted its training accuracy and loss every 150 images. This was because 20% of the total data was saved randomly to be tested through the model every time the set of 150 images was processed. This means that every time a set of training images (150) was used, the program tested the validation data of all 45 image sets, leading each image set to validate using the same 1440 images for all of them. Thus, the orange line representing validation accuracy and validation loss used 1440 of the same images every time and corresponded to more training data being input over time.

For the large image recognition algorithm, the training loss of our models declined to near 0%, whereas the validation loss ended up at around 17% (Figure 7A). The output of this algorithm was then fed into the small image recognition algorithm. The training loss was 0% and the validation loss ended up at about 16% (Figure 7B). Both algorithms had accuracies of 100% on the training data and ~98% on the validation data.

**Figure 8.**
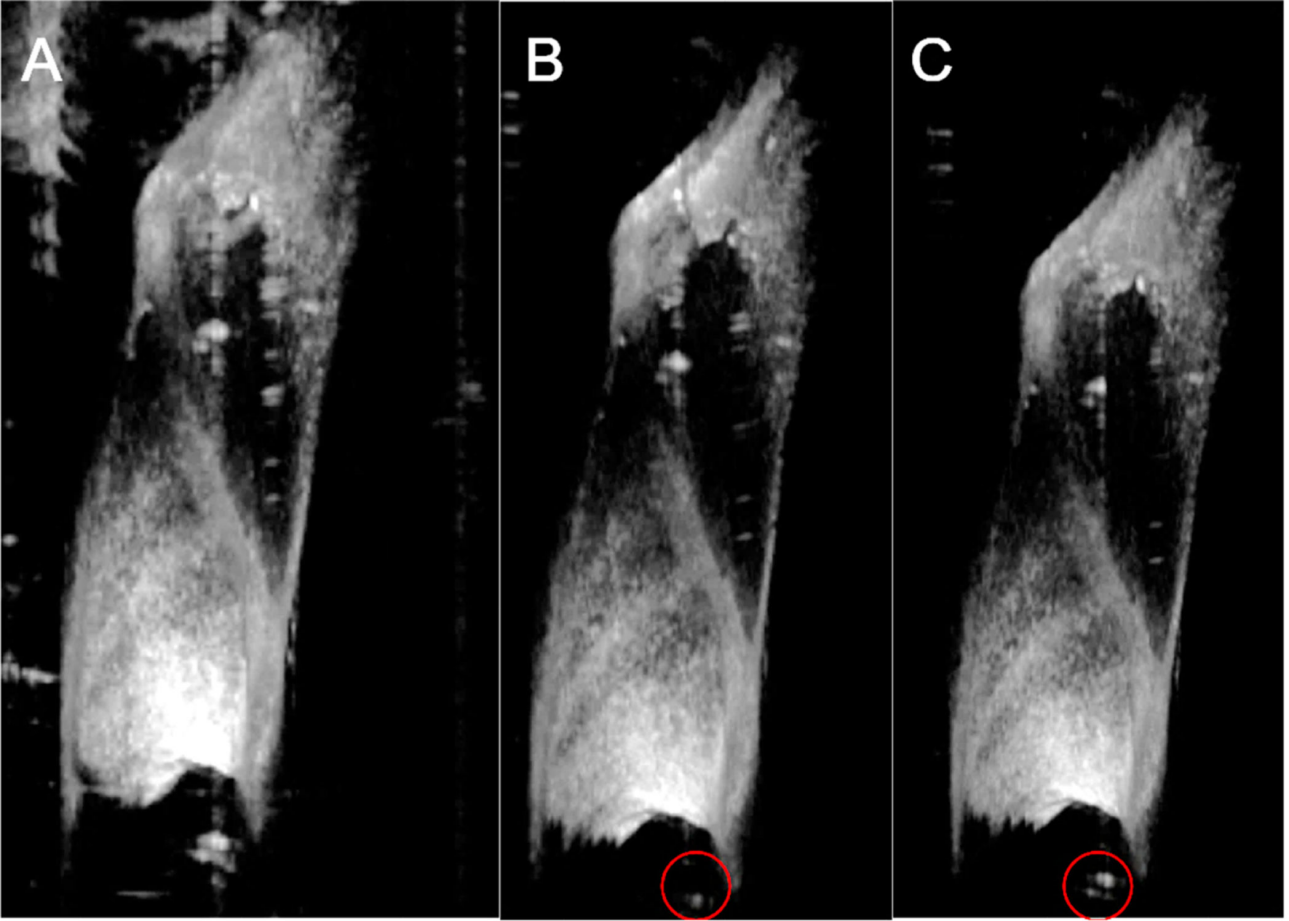
A representative 3D image of the TM before and after segmentation. (**A**) The original image. Note artifacts in the upper left. (**B**) The image after segmentation of the TM using the overfit algorithm from Figure 7B. (**C**) The image after segmentation of the TM using the algorithm from Figure 7C. The images in (**B**,**C**) are quite similar, but there is a little more artifact in (**C**) (red circle).

These data mean that the final output of the two-stage algorithm, as shown in Figure 7B, was quite good. However, the validation loss indicated overfitting. We kept this because, on a practical basis, it was not so overfitted as to adversely affect the functional output of the algorithm. In fact, we purposefully kept the algorithms slightly overfit, as we recognized that the additional training cycles led to improved TM detection by eye, even though this was not represented in these plots. To demonstrate this, we re-created the small image recognition algorithm using only 25% of the data (Figure 7C). After completing all of the training, the accuracy and training loss were similar to what was found using the full training set (i.e., Figure 7B), but the validation loss was only ~10%. We then visually inspected the images that were output using new data and found that the overfitted model had slightly better artifact removal (Figure 8).

## 4. Discussion

OCT provides a unique non-invasive way to assess human tissues. It offers the benefits of ease of use within a routine clinical setting and there are no known safety concerns. However, because swept-source laser light is used for imaging, artifacts caused by saturation of the photodetectors by reflected light or electrical noise within the high-speed analog-to-digital sampling system can obscure the image. Furthermore, because the high resolution of OCT provides near-cellular resolution, only a section of the tissue being studied can be imaged at once. Finally, OCT identifies multiple structural layers, including surface and sub-surface structures. These three issues (the presence of artifacts, the small field of view, and the long depth of view) can make it difficult for physicians to clinically interpret an OCT image. Here, we presented a technique to automatically segment out the TM from OCT images of the human ear. It removes artifacts and subsurface structures that are not the TM and then labels the TM specifically. Thus, the physician is presented with a clear and obvious OCT view of just the TM, which will allow the identification of pathology that is not detectable by routine otoscopy.

While neural-network-based automated segmentation of CT images of the human ear has been carried out before [37–40], a similar approach for segmentation of OCT images of the human ear is novel. Automatic segmentation is particularly important for OCT since provides a small field of view compared to CT or MRI, which can image the entire body. To provide OCT coverage of an entire structure, multiple 3D images from multiple angles might be needed. Once the structure of interest has been segmented out from each image, the 3D images could then be stitched together to provide a comprehensive analysis of the area of interest. For example, each of our microscopic OCT images can image about 2/3rds of the TM. Now that the TM can be segmented, we could envision a future where the user slowly moves the imaging probe around in a circle, collecting many overlapping 3D volumes, and then the algorithm automatically cleans them and stitches them together. In this way, we expect to collect large, high-resolution 3D images of the ear in clinic.

### 4.1. Deep Learning and Model Limitations

We chose to use deep learning for our algorithm because of its potential to revolutionize medical imaging for disease diagnosis [35,41,42]. Furthermore, we needed our algorithm to be rigorous and dynamic, so that, regardless of the section of the TM that was being imaged, the TM could be automatically segmented. We used TensorFlow to create most of the neural network, but the key to obtaining accurate results was the use of two sequential classification steps, each with distinct object detection algorithms.

As shown, our algorithm does a good job of removing most artifacts within the image. However, sometimes, the artifacts overlap or are very close to the TM, creating a conjunction of pixels that appears as one single object. Thus, most but not all artifact is removed. In the future, we may consider adding a third neural network with a separate image recognition algorithm specifically to identify and remove these types of artifacts, which tend to be vertical lines that originate from strong tissue reflections. While this may improve the image somewhat, it would also increase the processing time. Furthermore, it is unlikely to improve the image substantially enough to add further value to the clinician.

Most of the time creating this algorithm was spent manually classifying the objects. The object-detection algorithms created thousands of images from each 3D image. However, the first step was to optimize the large-object-detection algorithm so that it best recognized the TM as one single object but did not include nearby artifacts. This tradeoff required lowering the threshold value of the object detection algorithm so that the entire TM was recognized as one object instead of being broken up into several smaller objects. However, doing this led to more artifacts within the TM image. As described in the methods section, we handled this by adding the second stage to the algorithm, using more accurate sorting methods for the smaller object area of the TM. Hardware improvements to reduce the number and intensity of the artifacts within the original image would improve the accuracy of this algorithm and speed training of future iterations of this algorithm.

### 4.2. The Future: Automated Diagnosis of Ear Pathology

This algorithm to segment out the normal TM is the first step in the automatic detection of pathologic TMs. We anticipate that training the algorithm on images collected from patients with diseases, such as a tympanic membrane perforation, a retraction pocket, a cholesteatoma, or a tumor, will permit artificial intelligence to enhance, and perhaps even automate, the diagnosis of ear disease. Ultimately, we also plan to expand this algorithm to not only segment the TM but also other structures visible within the volume stack, including the ossicles and the cochlea. This will expand the number of diseases that AI-enhanced OCT could detect. Our goal is for an inexperienced clinician to be able to image their patient’s ear and for the software to automatically provide a tentative diagnosis with high accuracy.

## 5. Conclusions

Automatic segmentation is a core component needed to use AI to analyze 3D medical images. This algorithm demonstrates that deep learning can segment out the TM from within an artifact-filled OCT volume stack of images. This will be critical to the successful clinical implementation of OCT technology to detect ear disease. It allows a physician to have their computer create a 3D model of the TM quickly and ultimately, which will permit disease detection. Furthermore, it allows for immediate dialogue with the patient about the status of their ear; thus, it provides a point-of-care diagnostic. This provides an additional example of how deep learning is a powerful tool for medical imaging.

## Data Availability

Data and code supporting the findings of this study are available online in our lab’s GitHub account (https://github.com/jso111/) Accessed on 16 September 2023.
